# Fucoxanthin Pretreatment Ameliorates Visible Light-Induced Phagocytosis Disruption of RPE Cells under a Lipid-Rich Environment via the Nrf2 Pathway

**DOI:** 10.3390/md20010015

**Published:** 2021-12-23

**Authors:** Yunjun Liu, Zixin Guo, Shengnan Wang, Yixiang Liu, Ying Wei

**Affiliations:** 1 College of Ocean Food and Biological Engineering, Jimei University, Xiamen 361021, China; lyj28528x@163.com (Y.L.); zzxxgguo@163.com (Z.G.); wsnynlfighting@163.com (S.W.); 2 Collaborative Innovation Center of Provincial and Ministerial Co-Construction for Marine Food Deep Processing, Dalian Polytechnic University, Dalian 116034, China; 3 The Department of Food Engineering, China National Research Institute of Food & Fermentation Industries Corporation Limited, Beijing 100015, China

**Keywords:** fucoxanthin, RPE cells, phagocytosis, Nrf2, visible light, docosahexaenoic acid

## Abstract

Fucoxanthin, a special xanthophyll derived from marine algae, has increasingly attracted attention due to its diverse biological functions. However, reports on its ocular benefits are still limited. In this work, the ameliorative effect of fucoxanthin on visible light and lipid peroxidation-induced phagocytosis disruption in retinal pigment epithelium (RPE) cells was investigated in vitro. Marked oxidative stress, inflammation, and phagocytosis disruption were evident in differentiated RPE cells following their exposure to visible light under a docosahexaenoic acid (DHA)-rich environment. Following pretreatment with fucoxanthin, however, the activated nuclear factor erythroid-derived-2-like 2 (Nrf2) signaling pathway was observed and, furthermore, when the fucoxanthin -pretreated RPE cells were irradiated with visible light, intracellular reactive oxygen species (ROS), malondialdehyde (MDA) levels and inflammation were obviously suppressed, while phagocytosis was significantly improved. However, following the addition of Nrf2 inhibitor ML385, the fucoxanthin exhibited no ameliorative effects on the oxidative stress, inflammation, and phagocytosis disruption in the RPE cells, thus indicating that the ameliorative effect of fucoxanthin on the phagocytosis of RPE cells is closely related to the Nrf2 signaling pathway. In conclusion, these results suggest that fucoxanthin supplementation might be beneficial to the prevention of visible light-induced retinal injury.

## 1. Introduction

The proliferation of electronic devices (such as computers, widescreen phones, and televisions) and diverse lighting products has led to a dramatic increase in the incidences of light-induced photochemical eye damage and has become a major cause of visual health problems in modern society [1,2]. As the site of visual imaging and visible light focusing, retinal tissue is a vulnerable target for photochemical damage [3]. Not only does the retina metabolize and function under hyperoxic conditions, but the outer segments of its photoreceptors are also rich in photosensitizer molecules and polyunsaturated fatty acids (PUFAs) [4,5]. When the eyes are exposed to natural or artificial light sources for long periods of time, retinas become vulnerable to oxidative stress, thus greatly increasing the risk of retinopathy [6,7,8]. It is believed that excessive illumination can result in photoreceptor apoptosis, disturbance in the blood-retinal barrier, and inflammatory infiltration in the retina [9,10]. It has also been suggested that light-induced retinal injury can initiate age-related macular degeneration (AMD), which is a major cause of vision deterioration and blindness in the elderly [4,11]. Consequently, the prevention of retinal photo-oxidative damage through dietary nutritional supplementation has become a significant research focus for food scientists and nutritionists.

As one of the main targets of retinal photo-oxidative damage, and due to their important role in maintaining the physiological function of the retina, retinal pigment epithelium (RPE) cells and the potential to improve vision by maintaining their health through dietary supplementation have garnered increasing scientific attention. There is considerable evidence to confirm that RPE cells act as part of the outer blood-retinal barrier (BRB), controlling the exchange of nutrients and waste products between choroidal vessels and photoreceptor cells, and thus supporting the survival and normal functioning of photoreceptor cells [12]. In fact, the recycling of the photoreceptor outer segments (POSs) damaged by oxidation is completed via the phagocytosis of RPE cells [13]. Lipid peroxidation of PUFAs in the POSs can subject RPE cells to intense oxidative stress [4] and, therefore, the inhibition of photo-oxidative damage in RPE cells through dietary antioxidants is increasingly emphasized in research. In one study, berry-derived anthocyanins were reported to efficiently scavenge intracellular reactive oxide species (ROS) and down-regulate the expression of vascular endothelial growth factor (VEGF) in RPE cells under visible light exposure [14]. In another, epigallocatechin-3-gallate, a polyphenolic compound found in green tea, displayed a regulatory role in ultraviolet light irradiation-induced autophagy in RPE cells [15], while quercetin-3-O-α-L-arabinopyranoside reportedly exhibited an inhibitory effect on blue light-induced cell apoptosis and inflammation in RPE cells [16]. However, few studies have investigated dietary active ingredients to ameliorate visible light and lipid peroxidation-induced phagocytic disorder in RPE cells. In addition, while the RPE is a polarized monolayer of highly differentiated epithelial cells in vivo, current in vitro RPE culture models are unable to preserve many of their specific properties or to reproduce the functional features and gene expression patterns that RPE exhibits in vivo [17].

Nuclear factor erythroid-derived 2-like 2 (Nrf2), also known as nuclear factor erythroid 2-related factor 2, plays an important role as a main antioxidant pathway in a variety of diseases [18]. Under oxidative or other stress conditions, Nrf2 in the cytoplasm is translocated to the nucleus, thus regulating antioxidant response element (ARE)-mediated phase II detoxification and the expression of antioxidant proteins/enzymes, including glutamate-cysteine ligase (GCL), heme oxygenase-1 (HO-1), and NAD(P)H: quinone oxidoreductase (NQO1) [19,20]. There is growing evidence that the inhibition of Nrf2 signaling pathway activation further aggravates oxidative damage in cells [21]. It was recently reported that, after pigmented rabbits were exposed to visible light, the oral administration of dietary polyphenols could reduce light-induced retinal oxidative stress and further up-regulate the expression level of HO-1 mRNA [22]. Therefore, the regulation of Nrf2, as an upstream signaling molecule, would make it an attractive candidate gene as a regulatory target for improving phagocytosis in RPE cells during photo-oxidative stress.

Fucoxanthin, a special xanthophyll derived from edible brown seaweeds and some microalgae, has attracted attention due to its biological functions and unique structural properties, including epoxide, allenic, and acetyl groups [23,24]. In our earlier studies, fucoxanthin supplementation was found to provide comparatively superior performance to lutein in protecting the retina against visible light-induced damage, both in vitro and in vivo [23]. However, the mechanisms by which fucoxanthin ameliorates visible light-induced retinal damage have not yet been demonstrated. Accordingly, the aim of this study is to investigate the preventive effect of fucoxanthin on visible light-induced phagocytic dysfunction of RPE cells and the underlying mechanisms. In order to better mimic the tissue properties of pigment epithelium, differentiated RPE cell monolayers were employed in the construction of in vitro evaluation models. Additionally, as the most abundant polyunsaturated fatty acid (PUFA) in the POSs, docosahexaenoic acid (DHA) was used to create the lipid-rich environment to which the RPE cells are exposed in vivo. Under this in vitro model, the effects of the intensity and duration of visible light exposure on oxidative damage and phagocytic function of RPE cells were observed. Subsequently, the mechanism by which fucoxanthin improves the phagocytosis of RPE cells through the Nrf2 signaling pathway was elucidated. This study, thus, provides a theoretical basis for the use of marine fucoxanthin to prevent visual impairment caused by prolonged light exposure.

## 2. Results

### 2.1. Cytotoxicity of Fucoxanthin to RPE Cells in a Lipid-Rich Environment

**Figure 1:**
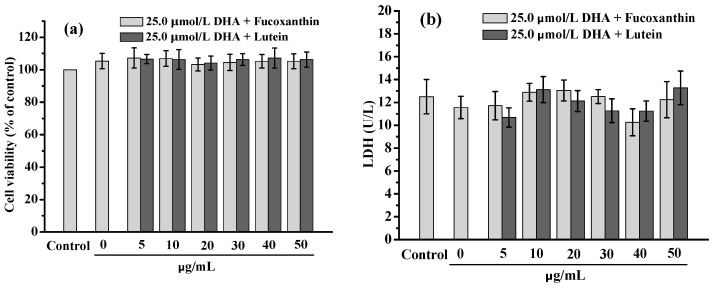
Cytotoxicity of fucoxanthin and lutein on RPE cells under a lipid-rich environment: (**a**) cell viability; (**b**) lactic dehydrogenase (LDH) levels. The DHA concentration was 25.0 μmol/L.

The cytotoxicity of fucoxanthin and lutein to RPE cells in a high-lipid environment was evaluated, with the results shown in Figure 1. When the medium contained 25.0 μmol/L of DHA, a slight proliferative effect was observed on the RPE cells after 24 h incubation, with no statistically significant difference between the fucoxanthin and lutein. It was therefore clear that under the condition of 25.0 μmol/L DHA, no obvious (*p* < 0.05) neither fucoxanthin nor lutein exerted a significant inhibitory effect on RPE cells’ proliferation when their dosages ≤50.0 μg/mL. Compared with the control group, there was also no significant difference (*p* < 0.05) in the lactic dehydrogenase (LDH) expression in all treatment groups. The LDH analysis further confirmed that 25.0 μmol/L DHA plus ≤50.0 μg/mL fucoxanthin or lutein exerted no cytotoxicity on the RPE cells.

### 2.2. Effects of Visible Light Exposure on Oxidative Damage in Differentiated RPE Cells

**Figure 2:**
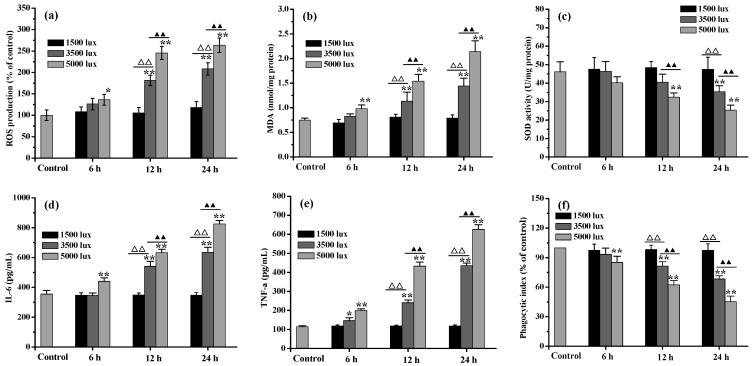
The effects of visible light exposure on oxidative damage, inflammation and phagocytosis in differentiated RPE cells: (**a**) intracellular ROS levels; (**b**) intracellular MDA levels; (**c**) SOD activity; (**d**) inflammatory factor IL-6; (**e**) inflammatory factor TNF-α; (**f**) phagocytic index. (* *p* < 0.05 and ** *p* < 0.01 vs. control; ^ΔΔ^ *p* < 0.01 means 1500 vs. 3500 lux; ^▲▲^ *p* < 0.01 means 3500 vs. 5000 lux).

The effects of different light exposure conditions on the oxidative damage to and phagocytic function of RPE cells were investigated, the results of which are presented in Figure 2. When the differentiated RPE cells were irradiated with 1500 lux visible light for 6–24 h, no obvious (*p* < 0.05) oxidative damage, inflammation, or phagocytic dysfunction were observed. However, when the light intensity was increased to 3500 or 5000 lux, visible light-induced injury was induced with prolonged light exposure. After 24 h light exposure under 3500 lux, the intracellular ROS and malondialdehyde (MDA) level had increased by approximately 1.46 and 1.05 times, respectively, while superoxide dismutase (SOD) activity had decreased by 29.85%. Compared with the control group, the inflammatory cytokines interleukin (IL)-6 (354.13 ± 25.44 pg/mL of control) and tumor necrosis factor-α (TNF-α) (114.13 ± 5.39 pg/mL of control) in the media were found to have increased to 632.36 ± 22.39 pg/mL and 432.36 ± 21.35 pg/mL, respectively, and a 37.64% loss was simultaneously observed in the phagocytic index. It is clear that under the more intense light exposure conditions and consequently more extensive oxidative damage, the phagocytic function of the RPE cells was further degraded. For example, when the RPE cells were exposed to light irradiation at 5000 lux for 24 h, their phagocytic index remained only 45.34 ± 5.68%. Considering the degree of oxidative damage and degradation of the phagocytic function of RPE cells, light exposure at 3500 lux for 24 h was employed in the subsequent experiments.

### 2.3. Fucoxanthin Pretreatment Activated the Nrf2 Signal Pathway in RPE Cells

**Figure 3:**
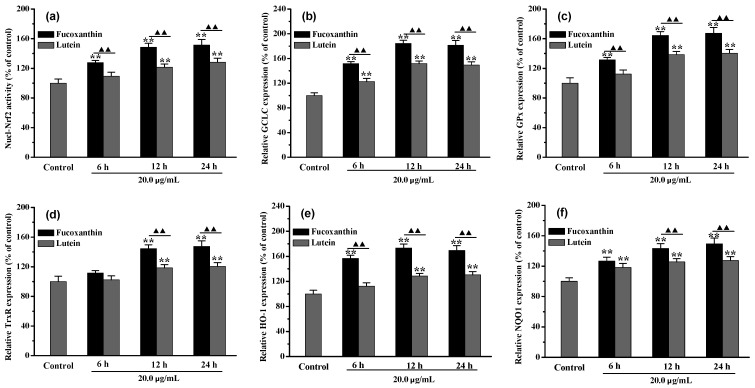
The effect of pretreatment time on Nrf2 signaling pathway activated by fucoxanthin in differentiated RPE cells. (**a**) Nucl-Nrf2 activity; (**b**) GCLC expression level; (**c**) GPx expression level; (**d**) TrxR expression level; (**e**) HO-1 expression level; (**f**) NQO1 expression level. (** *p* < 0.01 vs. control; ^▲▲^ *p* < 0.01 vs. lutein).

In this study, the results showed that adequate pretreatment time with fucoxanthin was beneficial in activating the antioxidant system of the RPE cells. As shown in Figure 3, after RPE cells were pretreated by fucoxanthin for 6–24 h, the expressions of nuclear Nrf2 (Nucl-Nrf2) protein and its regulated downstream antioxidant proteins or detoxification enzymes were investigated. When the RPE cells were co-incubated with 20.0 μmol/L fucoxanthin for 6 and 12 h (Figure 3a), it was found that, compared with the control group, Nucl-Nrf2 activity was increased by approximately 1.28 and 1.48 times, respectively. However, there was no further significant (*p* < 0.05) increase in Nucl-Nrf2 expression when the fucoxanthin pretreatment time was extended to 24 h. Interestingly, lutein, which is also a xanthophyll, performed worse (*p* < 0.01) than fucoxanthin in activating Nucl-Nrf2. After 12 h pretreatment, the Nucl-Nrf2 level in the lutein group had only increased 1.21 times. As shown in Figure 3b–e, similar phenomena were observed in the antioxidant proteins, including glutamate-cysteine ligase catalytic subunit (GCLC), glutathione peroxidase (GPx), thioredoxin reductase (TrxR), and HO-1, as well as the detoxification enzyme NQO1. The above results, thus, indicate that 12 h fucoxanthin pretreatment was sufficient to effectively activate the Nrf2 signaling pathway in the differentiated RPE cells.

### 2.4. Fucoxanthin Attenuated Visible Light-Induced Oxidative Stress and Phagocytosis Disorder in RPE Cells

**Figure 4:**
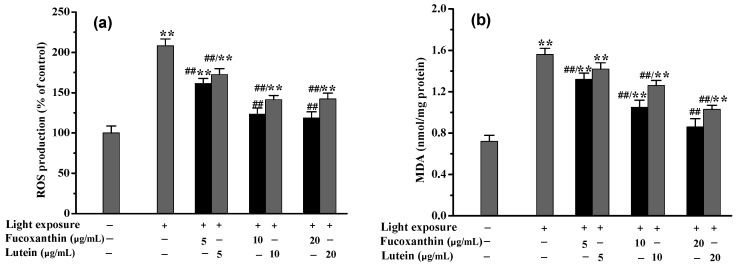

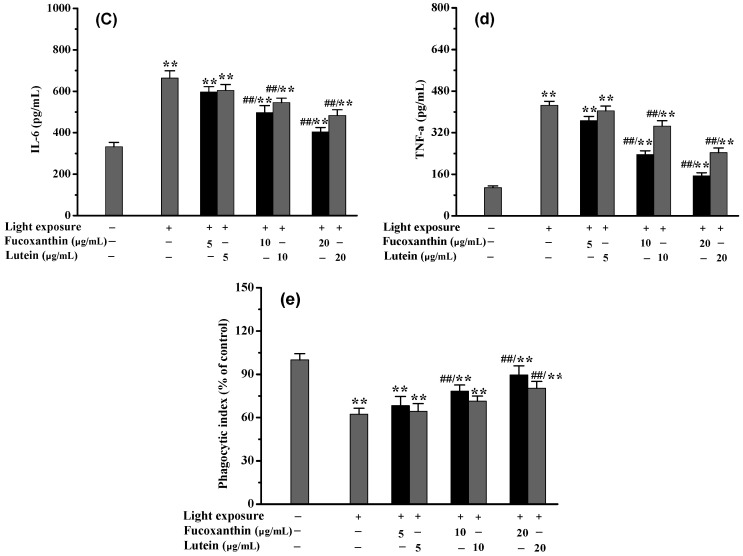
Inhibitory effects of fucoxanthin on oxidative damage and phagocytosis disorder in RPE cells induced by visible light: (**a**) intracellular ROS levels; (**b**) intracellular MDA levels; (**c**) inflammatory factor IL-6; (**d**) inflammatory factor TNF-α; (**e**) phagocytic indexes. (** *p* < 0.01 vs. control; ^##^ *p* < 0.01 vs. light exposure).

Following 12 h pretreatment with fucoxanthin, the differentiated RPE cells were subjected to visible light exposure at 3500 lux for 24 h. As shown in Figure 4, when the concentration of fucoxanthin was 5.0 μmol/L, both the intracellular ROS and MDA levels began to be effectively (*p* < 0.01) inhibited, although no obvious (*p* < 0.05) ameliorative effects on inflammation or phagocytosis were evident. However, when the fucoxanthin concentrations were further increased to 10.0 or 20.0 μmol/L, the oxidative stress and inflammatory response in the RPE cells were significantly (*p* < 0.01) ameliorated. Under the condition of 20.0 μmol/L fucoxanthin, the intracellular ROS level decreased from 208.24 ± 8.56% (model group) to 118.56 ± 7.68%, and, compared with the model group, the levels of MDA, IL-6 and TNF-α were reduced by 44.87, 39.16, and 63.89%, respectively. Simultaneously, the phagocytic index of RPE cells recovered from 62.36 ± 4.15% to 89.56 ± 6.36%.

### 2.5. Fucoxanthin Protected against Phagocytosis Disorder of RPE Cells via the Nrf2-Mediated Pathway

**Figure 5:**
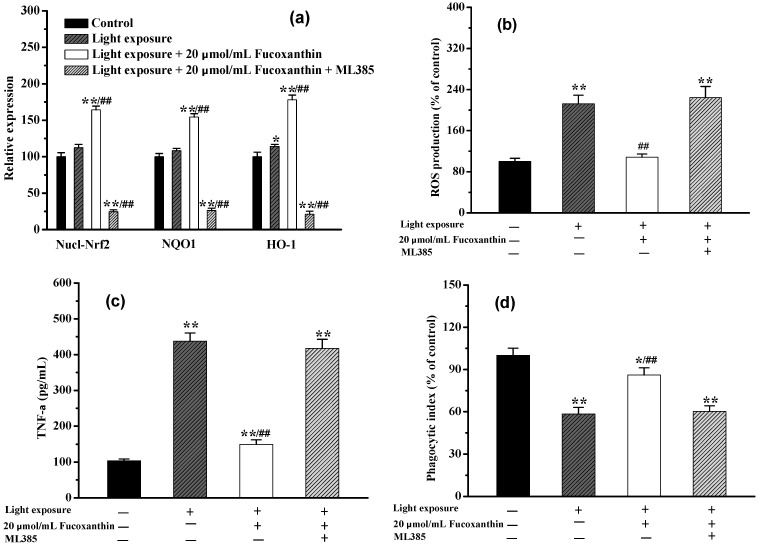
Ameliorative effects of fucoxanthin on phagocytosis disorder in RPE cells via the Nrf2 signal pathway: (**a**) the expressions of Nucl-Nrf2, NQO1, and HO-1 when RPE cells were treated with fucoxanthin or fucoxanthin +ML385; (**b**) ROS production when RPE cells were treated with fucoxanthin or fucoxanthin + ML385; (**c**) TNF-α levels when RPE cells were treated with fucoxanthin or fucoxanthin +ML385; (**d**) phagocytic indexes when RPE cells were treated with fucoxanthin or fucoxanthin +ML385. (* *p* < 0.05 and ** *p* < 0.01 vs. control; ^##^ *p* < 0.01 vs. light exposure).

In order to further elucidate the mechanism by which fucoxanthin provides protection against visible light-induced phagocytosis disorder of RPE cells, the Nrf2-mediated signaling pathway was investigated. As shown in Figure 5a, when the RPE cells were irradiated with visible light, the expression levels of Nucl-Nrf2 and its regulated NQO1 and HO-1 were slightly increased compared with those of the control group. Interestingly, when the RPE cells were administered with 20.0 μmol/L fucoxanthin (12 h pretreatment time plus 24 h light exposure time), the levels of Nucl-Nrf2, NQO1, and HO-1 were increased by approximately 1.64, 1.54, and 1.78, respectively, compared with those of the control group. Correspondingly, the intracellular ROS level was close to that of the control, while the TNF-α was reduced by 64.21%, and the phagocytic index recovered to approximately 86.04%. However, when the Nrf2 inhibitor ML385 was added at the same time as the fucoxanthin pretreatment of the RPE cells, the levels of Nucl-Nrf2, NQO1, and HO-1 after the light exposure were only 24.56, 26.21, and 21.16%, respectively, of those of the control group. Moreover, under the same ML385 pretreatment condition, the fucoxanthin did not exhibit ameliorative effects on the oxidative stress, inflammatory response, or phagocytosis disruption in the RPE cells. As shown in Figure 5b–d, there were obvious (*p* < 0.05) differences between the fucoxanthin +ML385 group and the model group at the intracellular ROS level, inflammatory factor TNF-α level, and the phagocytic index. Thus, it is clear that ML385 can effectively block activation of the Nrf2 signaling pathway, resulting in the inability of fucoxanthin to improve the phagocytosis of RPE cells.

## 3. Discussion

This work used a polarized RPE cell culture model exhibiting similar morphological and physiological characteristics to the intact RPE monolayer, including the apical microvilli, well-defined tight junctions, membrane transport capability, and melanocytic pigmentation [25]. In our previous studies, when unpolarized RPE cells were exposed to 3500 lux visible light plus 25.0 μmol/L DHA for 12 h, the phagocytic index remained at only approximately 50% [1]. However, a phagocytic index with 81.49 ± 4.36% was observed when the differentiated RPE cell monolayer was exposed to the same visible light and lipid environment, suggesting that the differentiated RPE cells exhibited greater tolerance to visible light exposure. Therefore, it was hypothesized that, compared with the conventional in vitro cell model, differentiated RPE cells would more effectively reflect the true physiological conditions of retinal photodamage.

The RPE is considered a major target for retinal photodamage, which has long been attributed to the intracellular accumulation of lipofuscin particles during the phagocytizing of POSs [1,26]. Upon irradiation with visible light, N-retinylidene-N-retinylethanol-amine (A2E), one of the bis-retinoids derived from lipofuscin, produces singlet oxygen and other ROS, thus becoming a source of oxidative stress in RPE cells [27,28]. Due to their abundance of PUFAs, lipid peroxidation of POSs occurs before being swallowed in the presence of light exposure [29]. Given the close contact between RPE and POSs, this lipid peroxidation appears to be an important cause of visible light-induced damage to RPE cells. The results of this present study further confirmed that under the condition of visible light plus DHA exposure, obvious oxidative injury occurred in the RPE cells. Numerous studies have indicated that excessive light exposure can not only lead to oxidative stress by triggering ROS generation but also induce retinal cell dysfunction. When Wistar rats were illuminated by white light-emitting diode light, disruption to the outer blood-retinal barrier, which is constructed by RPE cells, was observed [30]. Furthermore, excessive light exposure could result in increased vascular endothelial growth factor (VEGF) secretion in cultured RPE cells, thus increasing the risk of neovascularization and edema [31]. In our previous studies, cellular senescence was also discovered when RPE cells were subjected to visible light exposure [1], while, in this work, it was further found that intense visible light radiation could lead to the degradation of phagocytic functioning in differentiated RPE cells. It is, thus, apparent that the protection of RPE cells against visible light-induced oxidative damage and phagocytosis disorder within a PUFA-rich environment should be an important pathway for vision-protecting dietary nutrients.

Antioxidant activity has been shown to be one of the important ways in which dietary nutrients perform their vision-protective functions. For example, dietary flavonoids (such as fisetin, luteolin, and quercetin) and vitamins C and E are capable of protecting RPE cells against hydrogen peroxide (H_2_O_2_)- or *t*-butyl hydroperoxide (*t*-BOOH)-induced death by scavenging intracellular ROS [32]. Curcumin pretreatment was shown to display a protective effect on light-induced retinal degeneration in a rat model through antioxidant pathways [33], while malvidin-3-galactoside/glucoside, the characteristic component of blueberry anthocyanins, could reduce oxidative stress in RPE cells by decreasing the levels of ROS and MDA [34]. It was also reported that madecassoside, a major bioactive triterpenoid saponin, could attenuate H_2_O_2_-induced ROS and MDA production in RPE cells [35]. As mentioned above, RPE cells are highly susceptible to cell damage induced by lipid peroxidation in the body; however, as shown via the results in this work, fucoxanthin can effectively inhibit oxidative stress and inflammation induced by visible light plus DHA in RPE cells.

There is increasing evidence that the activation of endogenous cellular antioxidant systems is an important way in which dietary functional ingredients exert their antioxidant physiological activities. As a nuclear transcription factor, Nrf2 controls the expression and coordinated induction of a battery of defensive genes encoding detoxifying enzymes and antioxidant proteins, which are critically important mechanisms for cellular protection and cell survival [36]. Resveratrol pretreatment reportedly significantly restored the SOD activity and upregulated the protein and mRNA expressions of Nrf2 and HO-1 in rat brain [37] and, as a carotenoid, lycopene pretreatment has also been reported to be effective in enhancing the expressions of Nrf2 and HO-1 in rat brain, thereby exerting a neurocytoprotective function [38]. Furthermore, when RPE cells were pretreated with hesperetin for between 1 and 6 h, the effect on Nucl-Nrf2 activation was discovered to be treatment time-dependent [20]. Based on our present results, when RPE cells were pretreated with fucoxanthin for 6–24 h, the expressions of Nucl-Nrf2 and its regulated downstream antioxidant proteins (such as GCLC, GPx, TrxR, HO-1, and NQO1) increased progressively. In addition, the addition of Nrf2 inhibitor ML385 during the whole experiment was found to effectively inhibit the expressions of Nucl-Nrf2, NQO1, and HO-1, and no protective effect from fucoxanthin on the RPE cells was observed. Thus, it was apparent that fucoxanthin performed an ameliorative effect on the visible light-induced phagocytic disorder of the RPE cells via the Nrf2 signaling pathway.

## 4. Materials and Methods

### 4.1. Materials and Chemical Reagents

The fucoxanthin and lutein standards were purchased from Dexter Biotechnology Co., Ltd. (Chengdu, China). Dimethyl sulfoxide, Dulbecco’s modified Eagle’s/Ham’s F12 media, DHA, 3-(4,5-dimethylthiazol-2-yl)-2,5-diphenyl tetrazolium bromide (MTT), 2′,7′-dichlorofluorescin diacetate (DCFH-DA), blue fluorescent amine-modified microspheres (0.05 μm) and fetal bovine serum (FBS) were obtained from Sigma-Aldrich (MO, USA). Commercial test kits, including LDH (CAS: A020-2-2), MDA (CAS: A003-4-1), SOD (CAS: A001-3-1), and HO-1 (CAS: H246-1), were purchased from Nanjing Jiancheng Bioengineering Institute (Nanjing, Jiangsu, China). Penicillin, streptomycin, and Hanks’ balanced salt solution (HBSS) were obtained from Gibco Life Technologies (Grand Island, NY, USA). The IL-6 (CAS: SEKM-0007) and TNF-α (CAS: SEKM-0034)kits were purchased from Beijing Solarbio Science & Technology Co., Ltd. (Beijing, China). The nuclear extraction kit (CAS: ab113474), Nrf2 transcription factor assay kit (CAS: ab207223), GCLC (CAS: ab233632), NQO1 (CAS: ab184867) were purchased from Abcam Shanghai Trading Co., Ltd. (Shanghai, China). The antioxidant protein test kits, including GPx (CAS: BC1195) and TrxR (CAS: BC1155), were obtained from Beijing Solarbio Science & Technology Co., Ltd. (Beijing, China). The Nrf2 inhibitor ML385 was provided by Sigma-Aldrich (MO). All other reagents were analytical reagent-grade and purchased from the China National Pharmaceutical Industry Corporation Ltd. (Shanghai, China).

### 4.2. Cell Culture

The human RPE cell line, ARPE-19 (ATCC CRL–2302), was provided by the American Type Culture Collection (Mantissa, VA, USA). The cell cultures were maintained in Dulbecco’s modified Eagle’s/Ham’s F12 media (Invitrogen, Carlsbad, CA, USA), to which 10% fetal bovine serum (Sigma-Aldrich, St. Louis, MO, USA), 100 U/mL penicillin, and 100 mg/mL streptomycin were added at 37 °C under a humidified 5% CO_2_ atmosphere.

### 4.3. Cytotoxicity Evaluation

The MTT assay and LDH release assay were employed to measure cell viability and cell membrane integrity, respectively, according to a previous study [23]. Briefly, RPE cells were plated in 96-well plates at the concentration of 5 × 10^5^ cells/mL for incubation 48 h. The cells were then treated with serum-free F12 medium containing 25.0 μmol/L DHA and different concentrations of fucoxanthin or lutein. After 24 h incubation, the cell supernatant was collected for LDH analysis. Subsequently, 150 µL of serum-free F12 medium containing 0.50 mg/mL MTT was added into each plate well and incubated for 4 h. The medium was then replaced with dimethyl sulfoxide (DMSO), and the absorbance was measured at 570 nm. The LDH activity in the cell supernatant was determined using a commercial LDH kit, according to the manufacturer’s instructions.

### 4.4. Construction of Differentiated RPE Cell Monolayer

**Figure 6:**
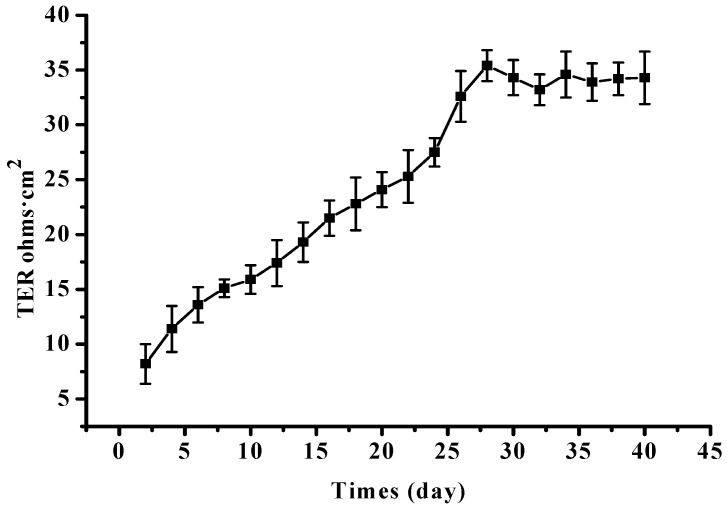
TER changes of the retinal pigment epithelium (RPE) cell monolayer during the cultivating processes.

The differentiated RPE cell monolayer was cultured as described in previous studies [39,40]. RPE cells at passage 4 were seeded onto transwell inserts with polyester membranes (6.5 mm diameter, 0.4 μm pores) from Corning Inc. (Corning, NY, USA), at a seeding density of 1 × 10^4^ cells per well (about 30,000 cells/cm^2^). In the apical and basolateral chambers, 200.0 and 600.0 μL of serum-free F12 media were added, respectively. The cultures were supplied with 5% CO_2_ in a humidified incubator (37 °C). Transepithelial electrical resistance (TER) was used to evaluate the differentiation degree of the RPE cell monolayer (Figure 6). It was deemed to be usable for subsequent light damage experiments when the differentiated RPE cell monolayer was cultured for 4 weeks, and the net TERs were ≥25.0 Ohm·cm^2^ [39].

### 4.5. Visible Light-Induced RPE Cell Injury In Vitro

Visible light- and lipid-induced RPE cell damage was performed based on our previous reports [41], with some modifications. Subsequent to the formation of the differentiated RPE cell monolayer, serum-free F12 medium containing 25.0 μmol/L of DHA was added into the apical chamber, whereafter the RPE cell monolayer was subjected to white light irradiation. The light intensities were set as 1500, 3500, or 5000 lx, with light exposure of 6, 12, or 24 h, after which periods oxidative damages to the RPE cells were assessed.

### 4.6. Fucoxanthin Pretreatment of RPE Cells

In order to effectively improve the resistance of RPE cells to oxidative stress, the effect of fucoxanthin pretreatment time on the Nrf2-regulated antioxidant system was observed. Fucoxanthin or lutein was dissolved with a small amount of DMSO and added to the serum-free F12 medium at a final concentration of 20.0 μmol/L. Subsequently, the medium (200.0 μL) was added into the apical basolateral chamber. After incubation for 6, 12, or 24 h, the expressions of Nrf2 protein, as well as its regulated downstream antioxidant proteins, were analyzed.

### 4.7. Protective Effect of Fucoxanthin against Visible Light-Induced Injury of RPE Cells

After 24 h pretreatment with fucoxanthin, the medium of the apical chamber was replaced by a serum-free F12 medium containing 20.0 μmol/L fucoxanthin and 25.0 μmol/L DHA. Thereafter, the cell monolayer was subjected to light irradiation for 24 h at the light intensity of 3500 lx. The protective effects of FUCO on visible light-induced phagocytic disorder and oxidative damage in the RPE cells were subsequently observed. In order to verify whether fucoxanthin had enhanced the ability of RPE cells to resist photo-oxidative stress through the Nrf2 pathway, the cells were pretreated with the Nrf2 inhibitor ML385 (10.0 μmol/L) for 24 h, and the inhibitor was also added during the light exposure [21].

### 4.8. Detection of ROS and MDA Content

The intracellular ROS and MDA levels were measured as described in previous studies [23,42], with some modifications. The medium in the apical chamber was replaced by new medium containing 25.0 μmol of DCFH-DA. After 1 h incubation at 37 °C, the supernatant containing DCFH-DA was removed, and the cell monolayer was washed with Hanks’ balanced salt solution (HBSS). The cell monolayer was then completely digested by RIPA lysis buffer, whereafter the cell lysate was added into a transparent black 96-well plate for fluorescence analysis. The fluorescence intensity was recorded using a microplate reader (Molecular Devices, San Jose, CA, USA) at 485 nm excitation and 530 nm emission. The MDA level in the cell lysate was measured according to the commercial kit’s instruction, which was based on the thiobarbituric acid reactive substance (TBARS) assay.

### 4.9. Detection of Intracellular SOD, HO-1, GCLC, GPx, NQO1, and TrxR Activity

After the cell monolayer was washed with HBSS and fully lysed by RIPA lysis buffer, the expressions of antioxidative enzymes, including SOD, HO-1, GCLC, GPx, NQO1, and TrxR, in the cell lysate were determined, according to the manufacturer’s protocols.

### 4.10. Detection of Inflammatory Cytokines

The inflammatory cytokines, including IL-6 and TNF-α, were measured using commercial ELISA kits following the manufacturer’s instructions.

### 4.11. Measurement of Nucl-Nrf2

The expression of Nucl-Nrf2 in the RPE cell monolayer was measured as described in a previous study, with some modifications [43]. Briefly, the differentiated RPE cell monolayer was treated with trypsin buffer at 37 °C for 10.0 min, whereafter the cells were collected and the nuclear proteins extracted using the nuclear extraction kit, according to the kit manufacturer’s instructions. The protein concentration of each sample was normalized to total protein content in the cell pellet using the Bradford assay. Next, 20.0 μg nuclear protein was added into the 96-well plate based on the instructions of the Nrf2 transcription factor assay kit. The optical density was recorded using a microplate reader (Molecular Devices, San Jose, CA, USA) at 450 nm.

### 4.12. Investigation of Phagocytosis of RPE Cells

The phagocytosis of the RPE cells was determined according to our previous studies [1,23], with some modifications. Following light irradiation, the supernatant in the apical chamber was replaced by a serum-free medium containing fluorescent microspheres (1 × 10^7^/mL). After 24 h incubation, the uningested microspheres were washed away with fresh serum-free medium, and, after being washed twice using HBSS, the cells were lysed by RIPA lysis buffer. Thereafter, the cell lysates were transferred to a black, clear-bottomed 96-well plate, and the fluorescence intensity was recorded using a microplate reader (Molecular Devices, San Jose, CA, USA) at excitation/emission 360/420 nm. The phagocytic index was expressed as fluorescence intensity and presented as a percentage relative to the control.

### 4.13. Statistical Analyses

All data were expressed as mean ± SD of at least three individual experiments. Statistical analyses were performed using SPSS Statistical 24 Software. Comparisons between groups were performed using one-way analysis of variance (ANOVA) with Duncan’s range tests. A normality test showed that all raw data displayed a normal distribution, while a variance test indicated that all groups exhibited equal variance. *p* < 0.05 (two-sided) was regarded as significant (*, *p* < 0.05; **, *p* < 0.01).

## 5. Conclusions

The results of this present study confirm that when differentiated RPE cells were exposed to both visible light (3500 lux or 5000 lux) and a PUFA-rich environment (25.0 μmol/L DHA) for 12–24 h, elevated oxidative stress and degenerative phagocytosis occur. However, fucoxanthin pretreatment at 20.0 μg/mL was found to effectively suppress the oxidative damage, inflammatory response, and phagocytosis disorder in RPE cells. Furthermore, the enhanced expression of Nucl-Nrf2 and the restored activity of detoxification enzymes such as HO-1 and NQO1 were simultaneously observed. Therefore, it is the conclusion of this work that the ameliorative effect of fucoxanthin on the phagocytosis of RPE cells should be closely related to the Nrf2 signaling pathway. However, recent studies have indicated that ARPE19 cells are different from natural RPE cells in terms of phenotype, including tight junction protein expression and pigment deposition. Therefore, in vivo animal experiments should be considered in future studies.
